# Effect of Sarcopenia on Mortality in Type 2 Diabetes: A Long-Term Follow-Up Propensity Score-Matched Diabetes Cohort Study

**DOI:** 10.3390/jcm11154424

**Published:** 2022-07-29

**Authors:** Jui-An Lin, Jin-De Hou, Szu-Yuan Wu

**Affiliations:** 1 Center for Regional Anesthesia and Pain Medicine, Wan Fang Hospital, Taipei Medical University, Taipei 11031, Taiwan; juian.lin@tmu.edu.tw; 2 Department of Anesthesiology, Wan Fang Hospital, Taipei Medical University, Taipei 11031, Taiwan; 3 Department of Anesthesiology, School of Medicine, National Defense Medical Center, Taipei 11490, Taiwan; jindehou805@gmail.com; 4 Department of Anesthesiology, School of Medicine, College of Medicine, Taipei Medical University, Taipei 11031, Taiwan; 5 Pain Research Center, Wan Fang Hospital, Taipei Medical University, Taipei 11031, Taiwan; 6 Department of Anesthesiology, School of Medicine, Shung Shan Medical University, Taichung City 40201, Taiwan; 7 Department of Anesthesiology, Shung Shan Medical University Hospital, Taichung City 40201, Taiwan; 8 Division of Anesthesiology, Hualien Armed Forces General Hospital, Hualien 97144, Taiwan; 9 Graduate Institute of Business Administration, College of Management, Fu Jen Catholic University, Taipei 24205, Taiwan; 10 Artificial Intelligence Development Center, Fu Jen Catholic University, Taipei 24205, Taiwan; 11 Department of Food Nutrition and Health Biotechnology, College of Medical and Health Science, Asia University, Taichung 41354, Taiwan; 12 Division of Radiation Oncology, Lo-Hsu Medical Foundation, Lotung Poh-Ai Hospital, Yilan 265501, Taiwan; 13 Big Data Center, Lo-Hsu Medical Foundation, Lotung Poh-Ai Hospital, Yilan 265501, Taiwan; 14 Department of Healthcare Administration, College of Medical and Health Science, Asia University, Taichung 41354, Taiwan; 15 Cancer Center, Lo-Hsu Medical Foundation, Lotung Poh-Ai Hospital, Yilan 265501, Taiwan; 16 Department of Management, College of Management, Fo Guang University, Yilan 262307, Taiwan

**Keywords:** type 2 diabetes, sarcopenia, nonsarcopenia, survival, prognostic factors

## Abstract

Purpose: The effect of sarcopenia on the survival of patients with type 2 diabetes remains unclear. Therefore, we designed a propensity score-matched population-based cohort study to compare the patients with diabetes with or without sarcopenia. Patients and Methods: We included patients with type 2 diabetes and categorized them into two groups according to whether they had sarcopenia and compared their survival; patients in the groups were matched at a ratio of 1:2. Results: The matching process yielded a final cohort of 201,698 patients (132,805 and 68,893 in the sarcopenia and nonsarcopenia diabetes groups, respectively) who were eligible for further analysis. According to both univariate and multivariate Cox regression analyses, the adjusted hazard ratios (aHRs; 95% confidence interval [CI]) of all-cause death for the sarcopenia diabetes group compared with the control group: 1.35 (1.33–1.38; *p* < 0.001). The aHRs (95% CIs) of all-cause death for those aged 41–50, 51–60, and >60 years (compared with those aged ≤40 years) were 1.53 (1.48–1.60), 2.61 (2.52–2.72), and 6.21 (5.99–6.45), respectively. The aHR (95% CI) of all-cause death for the male patients compared with the female patients was 1.56 (1.54–1.60). The aHRs (95% CIs) of all-cause death for those with adapted Diabetes Complications Severity Index (aDCSI) scores of 1, 2, 3, 4, and ≥5 (compared with an aDCSI score of 0) were 1.01 (1.00–1.14), 1.38 (1.35–1.42), 1.58 (1.54–1.63), and 2.23 (2.14–2.33), respectively. Conclusion: Patients with type 2 diabetes and sarcopenia had higher mortality than did those without sarcopenia.

## 1. Introduction

The incidence of type 2 diabetes mellitus in youth has increased in many countries since the early 1990s and is linked to an increase in childhood obesity [1,2,3,4]. Type 2 diabetes and its comorbidities are risk factors for vascular disease later in life and premature mortality [5]. Therefore, clinicians must identify and treat youth with this disease. Type 2 diabetes is a chronic disease not only in the older population but also in the younger population, and the prevalence of type 2 diabetes is increasing worldwide. Diabetes and its complications are leading causes of mortality in most countries [6,7,8,9]. In 2015, approximately 5 million individuals with diabetes aged between 20 and 79 years died, which is equivalent to one death every 6 s [6].

Sarcopenia is associated with insulin resistance, type 2 diabetes, and metabolic syndrome, which further increase the risks of cardiovascular disease and stroke [10,11]. Patients with severe sarcopenia were observed to have an almost four times increased risk of mortality compared with those without sarcopenia [12]. Sarcopenia is a syndrome characterized by the loss of muscle mass, strength, and performance [13,14]. Sarcopenia is associated with increased rates of functional impairment, disability, falls, and mortality [15]. The causes of sarcopenia are multifactorial and can include disuse, changes in endocrine function, chronic disease, inflammation, insulin resistance, and nutritional deficiencies [16]. The progressive loss of muscle mass in aging adults is a physical, functional, and metabolic catastrophe [17]. Sarcopenia predisposes individuals to osteopenia, weakness, loss of function, and frailty [18]. In addition, muscle tissue is increasingly being recognized as both an endocrine organ and a major contributor to whole-body insulin sensitivity [19].

Few studies with sufficient sample sizes and long-term follow-up periods have demonstrated an association of sarcopenia with incident all-cause mortality in patients with type 2 diabetes; moreover, previous studies have usually focused only on older individuals [20]. Sarcopenia occurs not only in older patients with type 2 diabetes but also in younger patients with type 2 diabetes. Therefore, we conducted a head-to-head propensity score-matched (PSM) study by using data from a nationwide diabetes cohort database to evaluate the association of mortality with sarcopenia in patients with diabetes. Early detection and management of sarcopenia might help increase overall survival (OS) in patients with type 2 diabetes if patients with diabetes and sarcopenia have higher mortality than do those without sarcopenia.

## 2. Patients and Methods

### 2.1. Data Sources and Study Cohort

We used data from January 2008 to December 2019 from Taiwan’s National Health Insurance (NHI) Research Database (NHIRD). The follow-up duration was from the index date to 31 December 2020. The NHIRD contains registration files and details regarding the original claims data of all NHI beneficiaries (i.e., approximately 27.38 million individuals). All NHIRD data, which are encrypted to protect beneficiaries’ privacy, include detailed outpatient and inpatient claims data including patient identification number; birth date; sex; diagnostic codes according to the *International Classification of Diseases, Ninth Revision, Clinical Modification* (*ICD-9-CM*) and the *International Classification of Diseases, Tenth Revision, Clinical Modification* (*ICD-10-CM*); treatment information; medical costs; dates of hospital admission and discharge; and date of death. All datasets can be interlinked through patient identification numbers. The study protocols were reviewed and approved by the Institutional Review Board of Tzu-Chi Medical Foundation (IRB109-015-B).

### 2.2. Participant Selection

Among a total of 540,698 patients with diabetes in the NHIRD, 68,893 with a diagnosis of sarcopenia and 411,101 without a diagnosis of sarcopenia were initially enrolled into the sarcopenia and nonsarcopenia groups, respectively, in the diabetes cohort. The inclusion and exclusion criteria and flow diagram for the selection procedure of the participants are presented as Supplemental Figure S1. Our definition of sarcopenia is according to the previous study from the Taiwan NHIRD [21]. In order to diminish the selection bias of the definition of sarcopenia, we only recorded the sarcopenia from the rehabilitation specialists, orthopedics, or family physicians. In Taiwan, the coding of sarcopenia was based on the previous Taiwan study [22]; sarcopenia was defined as the skeletal muscle mass index (SMI) of 2 standard deviations (SDs) or more below the normal sex-specific means for young persons. The index date was defined as the date of diabetes onset. The formula for SMI calculation is SMI = L3 skeletal muscle cross-sectional area (cm^2^)/height^2^ (m^2^) measured by computer tomography scan [1].

### 2.3. PSM and Covariates

After adjustment for confounders, we used a time-dependent Cox proportional hazards model to model time from the index date to all-cause death for the patients with diabetes and sarcopenia and those without sarcopenia. To reduce the effects of potential confounders when comparing all-cause death between the sarcopenia and nonsarcopenia diabetes groups, the participants were matched based on propensity scores. The matching variables used were age, sex, the adapted Diabetes Complications Severity Index (aDCSI; including the complication categories of retinopathy; nephropathy; neuropathy; and cerebrovascular, cardiovascular, peripheral vascular, and metabolic diseases) [23], Charlson comorbidity index (CCI) score, congestive heart failure, dementia, chronic pulmonary disease, rheumatic disease, liver disease, hemiplegia and paraplegia, renal disease, acquired immunodeficiency syndrome (AIDS), cancer, hypertension, hyperlipidemia, income levels, and urbanization levels. The aDCSI score (the sum of seven diabetes complications graded by severity as 0, 1, or 2; range: 0–13) was not matched but adjusted for in the Cox model. Comorbidities were determined according to *ICD-9-CM* codes in the main diagnosis of inpatient records or if the number of outpatient visits was ≥2 within 1 year. Continuous variables are presented as means ± standard deviations or medians (first and third quartiles), as appropriate. We matched the participants at a ratio of 2:1 by using the greedy method, matched with a propensity score within a caliper of 0.2 [24]. Matching is a common technique for selecting controls with identical background covariates as study participants, and it is done to minimize differences among study participants (that the investigator deems necessary to be controlled for).

### 2.4. Hazard Ratios of All-Cause Death between Patients with Diabetes

A Cox model was used to perform regression on the variables of all-cause death in the sarcopenia and nonsarcopenia diabetes groups, and a robust sandwich estimator was used to account for clustering within matched sets [25]. Even if PSM is performed, residual imbalance might still exist in a population [26,27]. Therefore, a multivariate Cox regression analysis should still be performed; hence, a multivariate Cox regression analysis was performed to calculate hazard ratios (HRs) with 95% confidence intervals (CIs) to determine whether factors such as sarcopenia, age, sex, aDCSI score, CCI score, income level, and urbanization level are potential independent predictors of all-cause death.

### 2.5. Statistical Analysis

All analyses were performed using SAS version 9.4 (SAS Institute, Cary, NC, USA). The matching procedure was implemented using PROC PSMATCH in SAS version 9.4 [28]. In a two-tailed Wald test, *p* < 0.05 was considered significant. Overall survival (OS) was estimated using the Kaplan–Meier method, and differences between the sarcopenia and nonsarcopenia diabetes groups were determined using the stratified log-rank test to compare survival curves (stratified according to matched sets) [29].

## 3. Results

### 3.1. PSM and Study Cohort

The matching process yielded a final cohort of 201,698 patients (132,805 and 68,893 in the sarcopenia and nonsarcopenia diabetes groups, respectively) who were eligible for further analysis; their characteristics are summarized in Table 1. The age distribution was balanced between the groups (Table 1). Age, sex, aDCSI score with complication categories, CCI score, congestive heart failure, dementia, chronic pulmonary disease, rheumatic disease, liver disease, hemiplegia and paraplegia, renal disease, AIDS, cancer, hypertension, hyperlipidemia, income level, and urbanization level were similar after head-to-head PSM between the groups, and no significant differences in the variables were observed between the groups. The aDCSI scores were significantly higher in the sarcopenia diabetes group than in the nonsarcopenia diabetes group. The primary endpoint of all-cause death in the sarcopenia diabetes group significantly varied from that in the nonsarcopenia group (*p* < 0.001; Table 1).

### 3.2. Prognostic Factors for All-Cause Death after Multivariate Cox Regression Analysis

**Figure 1:**
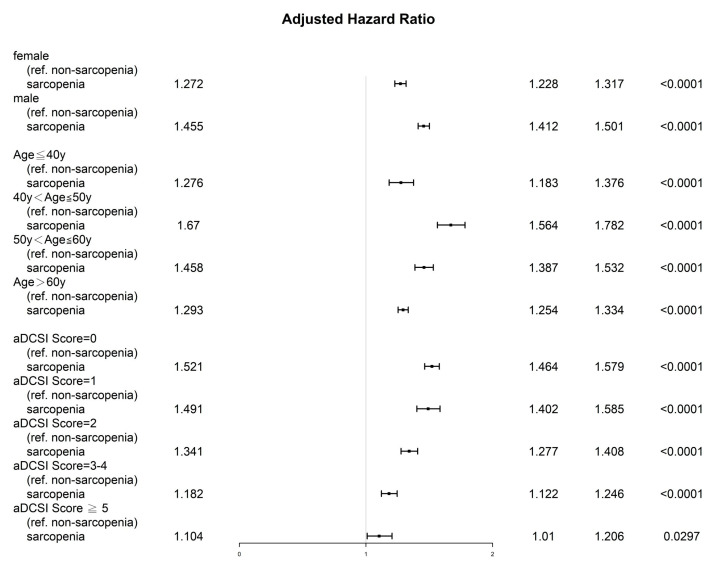
Sensitivity analysis of sex, age group, and adapted Diabetes Complications Severity Index scores performed using the inverse probability of treatment weighting for all-cause death in patients with diabetes with and without sarcopenia. Abbreviations: aHR, adjusted hazard ratio; y, years old; aDCSI, adapted Diabetes Complications Severity Index; CI, confidence interval; ref., reference group. All covariates presented in Table 2 were adjusted.

The results of multivariate Cox regression analysis indicated that the sarcopenia diabetes group exhibited less favorable prognostic factors for OS (Table 2). No significant differences were observed in explanatory variables, except for age ≥ 40 years, male sex, and aDCSI score ≥1. In multivariate Cox regression analysis, the aHR (95% CI) of all-cause death for the sarcopenia diabetes group compared with the control group was 1.35 (1.33–1.38, *p* < 0.001). The aHRs (95% CIs) of all-cause death for those aged 41–50, 51–60, and >60 years (compared with those aged ≤40 years) were 1.53 (1.48–1.60), 2.61 (2.52–2.72), and 6.21 (5.99–6.45), respectively (Table 2). The aHR (95% CI) of all-cause death for male patients compared with female patients was 1.56 (1.54–1.60). The aHRs (95% CIs) of all-cause death for those with aDCSI scores of 1, 2, 3, 4, and ≥5 (compared with an aDCSI score of 0) were 1.01 (1.00–1.14), 1.38 (1.35–1.42), 1.58 (1.54–1.63), and 2.23 (2.14–2.33), respectively. The results of the sensitivity analysis of sex, age groups, and aDCSI scores determined using the inverse probability of treatment weighting for all-cause death in the patients with diabetes with and without sarcopenia are presented as a forest plot in Figure 1. The aHRs (95% CIs) for the sarcopenia diabetes group were significantly associated with higher mortality compared with the control group, regardless of age group, sex, or aDCSI scores.

### 3.3. Kaplan–Meier Survival Curve of the Sarcopenia and Nonsarcopenia Diabetes Groups

**Figure 2:**
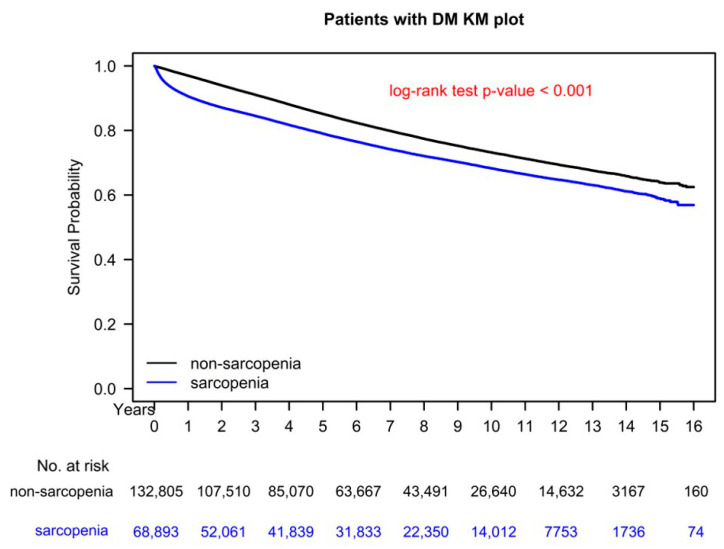
Kaplan–Meier overall survival curves for patients with diabetes with and without sarcopenia.

Figure 2 presents the survival curve (in terms of OS) obtained using the Kaplan–Meier method for the sarcopenia and nonsarcopenia diabetes groups. The 10-year OS rates for the two groups were 78.39% and 72.64%, respectively (*p* < 0.001).

## 4. Discussion

Sarcopenia is an independent risk factor for OS in many types of chronic diseases including cancers [30,31,32,33,34]; however, a study including a large sample size and long-term follow-up period has not investigated the effect of sarcopenia on OS in patients with diabetes; this is the largest and longest follow-up diabetes cohort study to estimate the association of OS with sarcopenia in patients with diabetes. In the current study, we used the head-to-head PSM design, mimicking a randomized controlled trial (RCT), to eliminate the potential bias of all-cause death in the diabetes cohort with or without sarcopenia. To reduce the effect of the severity of diabetes on OS, we matched the severity of diabetes by aDCSI score based on the complication category and adjusted aDCSI scores to determine the effect of sarcopenia on OS in the patients with diabetes. Our results revealed that the aHR (95% CI) of all-cause death for the sarcopenia diabetes group compared with the control group was 1.35 (1.33–1.38; *p* < 0.001). The sensitivity analysis indicated that aHRs (95% CIs) for the sarcopenia diabetes group were significantly associated with higher mortality compared with the control group, regardless of age group, sex, or aDCSI score.

How sarcopenia contributes to mortality in patients with diabetes remains unclear. Patients with diabetes have exhibited a significantly higher risk of sarcopenia [35,36,37]. In addition to age, the presence of diabetes is a determining factor for all-cause mortality, which exerts a profound effect on the onset and progression of sarcopenia. A potential mechanism might be the insufficient action of insulin. Insulin is an anabolic hormone that can accelerate human skeletal muscle protein synthesis [38]. Another mechanism underlying the high mortality in patients with diabetes and sarcopenia might be the linkage of sarcopenia with decreased protein synthesis and increased protein degradation caused by decreased antioxidant defenses [39], increased oxidative stress [39], and inflammation [40], leading to mortality [41]; moreover, these factors are associated with atherosclerosis [42,43,44,45] and contribute to mortality caused by cardiovascular or cerebrovascular diseases. Sarcopenia, especially low muscle strength, has been associated with blood pressure variability and cardiovascular event risk [42,43,44,45]. In addition, sarcopenia has reportedly been associated with a high risk of malnutrition [46,47], which aggravates sarcopenia, resulting in an increased mortality rate.

After PSM, all the covariates were balanced between the case and control groups. We used a well-designed PSM design to ensure the homogeneity of the potential bias of all-cause death. Performing an RCT to evaluate mortality in patients with diabetes with or without sarcopenia is difficult because sarcopenia cannot be treated through a tangible intervention [48]. Striking a balance among the confounding factors of mortality in patients with diabetes with and without sarcopenia (i.e., the case and control groups, respectively)—a main requirement of RCT design—is impossible [48]. Therefore, a PSM-based design, such as that used in the current study, can resolve this problem by maintaining balance among the confounding factors of the case and control groups in the absence of bias; moreover, PSM is currently the recommended standard tool for estimating the effects of covariates in studies where any potential bias may exist [24,49]. Although the main advantage of the PSM methodology is the more precise estimation of covariate effects, PSM cannot control for factors not accounted for in the model; moreover, PSM is predicated on an explicit selection bias of those who could be matched, meaning that individuals who could not be matched are not part of the scope of inference. Our study is the first to use a well-designed PSM design mimicking an RCT to investigate the effect of sarcopenia on OS in patients with diabetes.

The multivariate Cox regression results revealed that older age, male sex, and high aDCSI score were significantly poor prognostic factors for the patients with sarcopenia (Table 2); this finding is compatible with those of previous studies [50,51,52]. Other potential confounding factors were not significantly different for all-cause death between the two groups due to the use of a well-matched PSM design that lacked residual imbalance [26,27]. In general, female life expectancy exceeds male life expectancy [50]. Therefore, higher mortality was observed in the male patients with diabetes than in the female patients in the current study. The mortality rates increased steeply with advancing age in the patients with diabetes in our study; this finding is similar to that of a previous study [51]. In addition, our study findings are compatible with those reported by Wicke et al., who indicated that the aDCSI can be used as a severity measure for diabetes complications because it is correlated to and predicts mortality [52].

The forest plots of aHRs for all-cause death comparing different ages, sexes, and aDCSI scores among the diabetes patients with or without sarcopenia were analyzed (Figure 1). The effect of sarcopenia on OS was larger in those with lower aDCSI scores, male patients, and younger patients than in those with higher aDCSI scores, female patients, and older patients. The findings of sensitivity analysis (Figure 1) indicated that in the sarcopenia diabetes group, OS was poor in the younger patients, those with lower aDCSI scores, and male patients. Our study demonstrated that physicians should pay more attention to younger patients with diabetes and sarcopenia because of their higher mortality rates compared with those of older patients with diabetes and sarcopenia. Our findings are different from those reported by Takahashi et al., who enrolled only older patients (aged > 60 years) and indicated that sarcopenia was associated with incident all-cause mortality in older outpatients with type 2 diabetes [20]; moreover, they included only a small sample size (396 patients with diabetes) and short-term follow-up period (mean: 40.5 months) [20]; however, higher aHRs of all-cause death compared with those in the patients without sarcopenia were noted in our study. In the future, physicians should pay more attention to male patients, younger patients, and those with low aDCSI scores among patients with diabetes and sarcopenia because of the risk of higher mortality in these patients; this is the first study to indicate that male patients, younger patients, and those with lower aDCSI scores among the patients with diabetes and sarcopenia might be the susceptible population with high aHRs of all-cause death compared with older patients, female patients, and those with higher aDCSI scores.

The strength of our study is the inclusion of the largest and long-term follow-up comparative cohort study to estimate the primary endpoint of all-cause death in patients with or without sarcopenia. The covariates between the two groups were homogenous for the case and control groups. No bias was noted between the two groups through PSM (Table 1). Few studies have estimated OS in patients with type 2 diabetes and sarcopenia; this is the first study to report poorer OS in patients with sarcopenia than in those without sarcopenia, regardless of age, sex, and aDCSI score. In addition, this is the first study to indicate that the effect of sarcopenia on OS was larger for those with lower aDCSI scores, male patients, and younger patients compared with those with higher aDCSI scores, female patients, and older patients. In the future, prevention, correction, or treatment of sarcopenia would be valuable for not only improving the quality of life but also prolonging the survival of patients with type 2 diabetes. Our findings can be valuable for establishing health policies in the future; moreover, physicians should educate patients with sarcopenia to more aggressively overcome sarcopenia based on our results, especially male patients, younger patients, and those with lower aDCSI scores.

This study has some limitations. First, because all the patients with and without sarcopenia were enrolled from an Asian population, the corresponding ethnic susceptibility compared with that of a non-Asian population remains unclear; hence, our results should be cautiously extrapolated to non-Asian populations, however, no study has demonstrated significant differences in sarcopenia between Asian and non-Asian patients with diabetes. Second, although the aDCSI (severity of diabetes) has been matched, the therapy of carbohydrate metabolism disorders and whether patients were adherent to this therapy during the observation period were unavailable [53,54]. Third, the diagnoses of all comorbid conditions were based on *ICD-9-CM* or *ICD-10-CM* codes. Nevertheless, the NHIRD reviews charts and interviews patients to verify the accuracy of the diagnoses, and hospitals with outlier chargers or practices may be audited and subsequently be heavily penalized if malpractice or discrepancies are identified. Accordingly, to obtain crucial information on population specificity and disease occurrence, a large-scale RCT comparing carefully selected patients having sarcopenia and no sarcopenia would be necessary but might be difficult to be performed. Finally, the NHIRD does not contain information on dietary habits, which may be a risk factor for all-cause death. Despite these limitations, a major strength of this study is the use of a nationwide population-based registry with detailed baseline information. Lifelong follow-up was possible through the linkage of the registry with the national Cause of Death database. Considering the magnitude and statistical significance of the observed effects in the current study, the limitations are unlikely to affect our conclusions.

## 5. Conclusions

Patients with type 2 diabetes and sarcopenia had higher mortality than did those without sarcopenia, irrespective of age, sex, and diabetes severity. The effect of sarcopenia on OS was larger in those with lower aDCSI scores, male patients, and younger patients with diabetes compared with those with higher aDCSI scores, female patients, and older patients with diabetes.

## Figures and Tables

**Table 1:** Characteristics of patients with diabetes with and without sarcopenia (after propensity score matching at a ratio of 2:1).

	Nonsarcopenia		Sarcopenia		SMD
	N = 132,805		N = 68,893		
	N	%	N	%	
**Age** (mean ± SD)	59.92 ± 15.37		60.44 ± 14.61		0.0350
	60.00 (51.00, 71.00)		61.00 (51.00, 71.00)		
**Age Groups**	132,805		68,893		0.0120
Age ≤ 40 y	33,099	24.92%	16,979	24.65%	
40 y < Age ≤ 50 y	33,788	25.44%	17,356	25.19%	
50 y < Age ≤ 60 y	30,430	22.91%	15,805	22.94%	
Age > 60 y	35,488	26.72%	18,753	27.22%	
Sex	132,805		68,893		0.0010
Female	70,870	53.36%	36,813	53.44%	
Male	61,935	46.64%	32,080	46.56%	
**aDCSI Score**	132,805		68,893		1.0240
0	71,455	53.80%	32,071	46.55%	
1	24,920	18.76%	14,632	21.24%	
2	20,103	15.14%	11,344	16.47%	
3–4	13,049	9.83%	8466	12.29%	
≥5	3278	2.47%	2380	3.45%	
**aDCSI with complication categories**					
Retinopathy	7812	5.88%	3979	5.78%	0.005
Nephropathy	18,016	13.57%	10,240	14.86%	0.037
Neuropathy	11,619	8.75%	9211	13.37%	0.148
Cerebrovascular	13,500	10.17%	8936	12.97%	0.088
Cardiovascular	33,688	25.37%	20,630	29.94%	0.102
Peripheral Vascular Disease	4290	3.23%	2699	3.92%	0.037
Metabolic Disease	1273	0.96%	906	1.32%	0.034
**CCI Score**	132,805		68,893		0.0150
0	61,097	46.01%	31,405	45.59%	
≥1	71,708	53.99%	37,488	54.41%	
**Comorbidities**					
Congestive Heart Failure	8898	6.70%	4643	6.74%	0.002
Dementia	3558	2.68%	2174	3.16%	0.028
Chronic Pulmonary Disease	25,990	19.57%	13,966	20.27%	0.018
Rheumatic Disease	3380	2.55%	2129	3.09%	0.033
Liver Disease	26,766	20.15%	14,079	20.44%	0.007
Hemiplegia and Paraplegia	2332	1.76%	1703	2.47%	0.005
Renal Disease	6954	5.24%	3720	5.40%	0.007
AIDS	55	0.04%	22	0.03%	0.005
Cancer	15,944	12.01%	10,174	14.77%	0.021
Hypertension	74,277	55.93%	39,906	57.92%	0.0400
Hyperlipidemia	56,211	42.33%	30,085	43.67%	0.0270
**Income levels (NTD)**	132,805		68,893		0.0520
Low income	1572	1.18%	1096	1.59%	
≤20,000	84,866	63.90%	43,144	62.62%	
20,001–30,000	27,059	20.37%	14,908	21.64%	
30,001–45,000	12,714	9.57%	6665	9.67%	
>45,000	6594	4.97%	3080	4.47%	
**Urbanization**	132,805		68,893		0.1180
Rural	38,304	28.84%	23,658	34.34%	
Urban	94,501	71.16%	45,235	65.66%	
					***p*** **Value**
**Follow up**, Years (**mean** ± SD)	7.96 ± 4.62		7.61 ± 4.24		
**Follow up**, Years **Median** (IQR; Q1, Q3)	7.72 (2.68, 9.13)		7.46 (2.08, 9.19)		
**All-cause Death**	132,805		68,893		<0.0001
No	107,317	80.81%	51,560	74.84%	
Yes	25,488	19.19%	17,333	25.16%	

AIDS, acquired immune deficiency syndrome; CCI, Charlson comorbidity index; IQR, interquartile range; SD, standard deviation; NTD, New Taiwan dollar; N, number; y, years old; aDCSI, adapted Diabetes Complications Severity Index; SMD, standardized mean difference.

**Table 2:** Univariable and multivariable Cox proportional regression results for all-cause death of the propensity score-matched patients with diabetes.

	Crude HR (95% CI)		*p* Value	aHR * (95% CI)		*p* Value
**Sarcopenia (ref. No)**						
Yes	1.367	(1.34, 1.39)	<0.0001	1.356	(1.33, 1.38)	<0.0001
**Age** (ref. Age ≤ 40 y)						
40 y < Age ≤ 50 y	1.576	(1.51, 1.64)	<0.0001	1.539	(1.48, 1.60)	<0.0001
50 y < Age ≤ 60 y	3.182	(3.07, 3.3)	<0.0001	2.615	(2.52, 2.72)	<0.0001
Age > 60 y	9.72	(9.4, 10.05)	<0.0001	6.213	(5.99, 6.45)	<0.0001
Sex (ref. female)						
Male	1.664	(1.63, 1.7)	<0.0001	1.569	(1.54, 1.60)	<0.0001
**aDCSI Score** (ref.: aDCSI Score = 0)						
1	1.126	(1.09, 1.16)	<0.0001	1.011	(1.00, 1.14)	0.0442
2	2.635	(2.57, 2.7)	<0.0001	1.385	(1.35, 1.42)	<0.0001
3–4	3.72	(3.62, 3.82)	<0.0001	1.587	(1.54, 1.63)	<0.0001
≥5	6.245	(6, 6.5)	<0.0001	2.233	(2.14, 2.33)	<0.0001
**CCI** ≥ 1 (ref.: CCI = 0)	1.146	(0.59, 1.27)	0.4592	1.094	(0.75, 1.24)	0.3652
**Income (NTD)** (ref. Low income)						
≤20,000	0.733	(0.63, 1.12)	0.5907	0.749	(0.60, 1.08)	0.5892
20,001–30,000	0.654	(0.33, 1.08)	0.4981	0.656	(0.43, 1.09)	0.4870
30,001–45,000	0.617	(0.35, 1.18)	0.4672	0.621	(0.39, 1.16)	0.4562
>45,000	0.541	(0.33, 1.16)	0.3985	0.556	(0.32, 1.19)	0.3871
**Urbanization** (ref. rural)						
urban	0.733	(0.62, 1.05)	0.3095	0.904	(0.79, 1.09)	0.3087

aHR, adjusted hazard ratio; CCI, Charlson comorbidity index; NTD, New Taiwan dollar; y, years old; aDCSI, adapted Diabetes Complications Severity Index; CI, confidence interval; HR, hazard ratio; ref., reference group. * All covariates presented in Table 2 were adjusted.

## Data Availability

Data analyzed during the study were provided by a third party. We used data from the National Health Insurance Research Database (NHIRD). The authors confirm that, for approved reasons, some access restrictions apply to the data underlying the findings. The data utilized in this study cannot be made available in the manuscript, the supplementary files, or in a public repository due to the “Personal Information Protection Act” executed by Taiwan’s government, starting from 2012. Requests for data can be sent as a formal proposal to obtain approval from the ethics review committee of the appropriate governmental department in Taiwan. Specifically, links regarding contact info for which data requests may be sent to are as follows: http://nhird.nhri.org.tw/en/Data_Subsets.html#S3 and http://nhis.nhri.org.tw/point.html.

## Supplementary Materials

The following are available online at https://www.mdpi.com/article/10.3390/jcm11154424/s1.

## Abbreviations

HR	hazard ratio
aHR	adjusted hazard ratio
CI	confidence interval
RCT	randomized controlled trial
PSM	propensity score matching
*ICD-9-CM*	*International Classification of Diseases, Ninth Revision, Clinical Modification*
*ICD-10-CM*	*International Classification of Diseases, Tenth Revision, Clinical Modification*
OS	overall survival
CCI	Charlson comorbidity index
ESRD	end-stage renal disease
IQR	interquartile range
SD	standard deviation
NTD	New Taiwan dollar
N	number
y	years old
aDCSI	adapted Diabetes Complications Severity Index
SMD	standardized mean difference
NHI	National Health Insurance
NHIRD	National Health Insurance Research Database
