# Supplementary figures and images for: Effect of Sarcopenia on Mortality in Type 2 Diabetes: A Long-Term Follow-Up Propensity Score-Matched Diabetes Cohort Study
Source: J Clin Med. 2022 Jul 29;11(15):4424. doi: 10.3390/jcm11154424 (PMC9369839; doi:10.3390/jcm11154424)

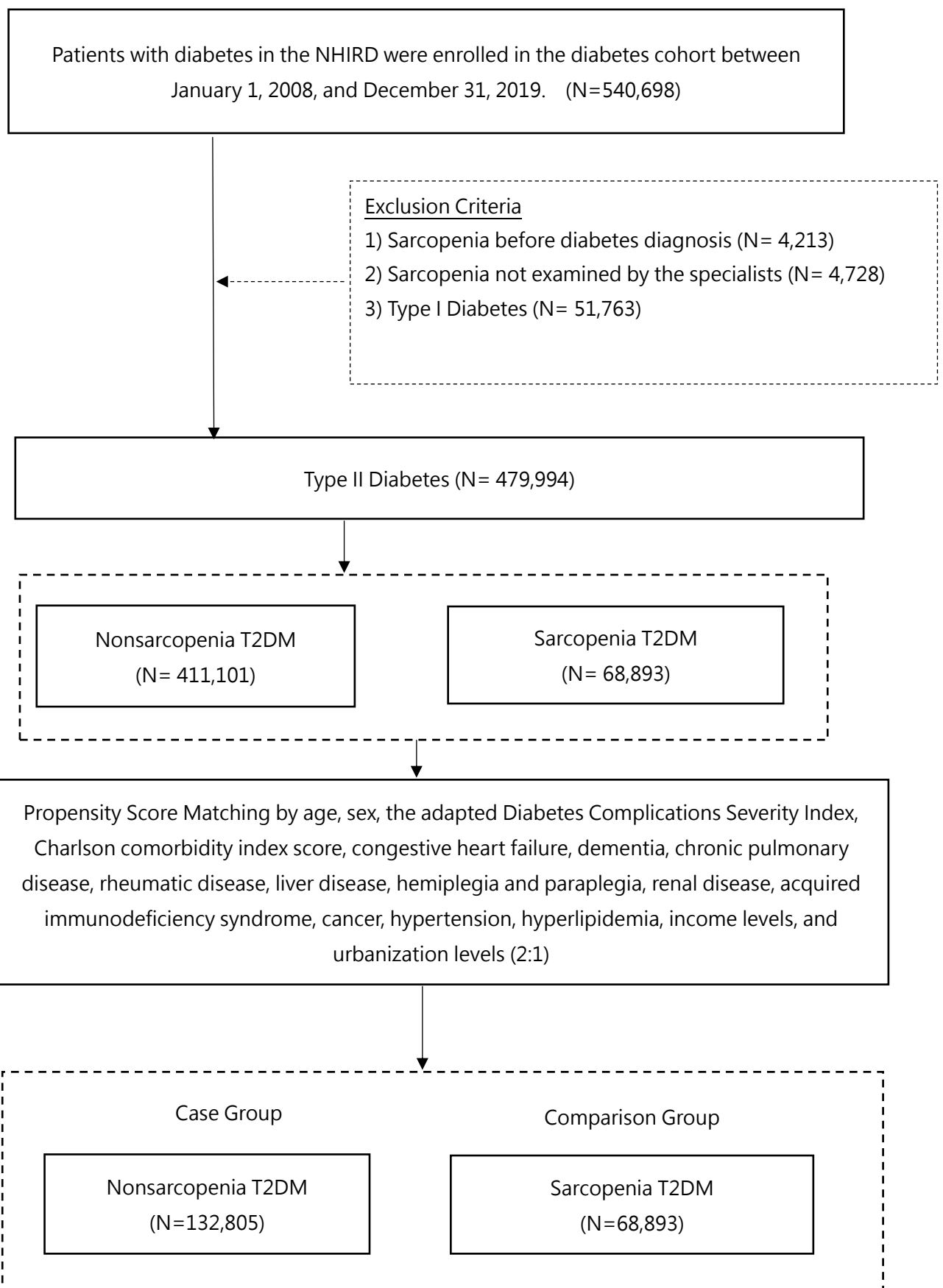

Supplement: Supplementary file 1 [file jcm-11-04424-s001.zip › jcm-1768433-supplementary.pdf]
